# Multi-Target Protective Effects of Gintonin in 1-Methyl-4-phenyl-1,2,3,6-tetrahydropyridine-Mediated Model of Parkinson’s Disease via Lysophosphatidic Acid Receptors

**DOI:** 10.3389/fphar.2018.00515

**Published:** 2018-05-23

**Authors:** Jong Hee Choi, Minhee Jang, Seikwan Oh, Seung-Yeol Nah, Ik-Hyun Cho

**Affiliations:** ^1^Department of Science in Korean Medicine and Brain Korea 21 Plus Program, Graduate School, Kyung Hee University, Seoul, South Korea; ^2^Department of Convergence Medical Science, College of Korean Medicine, Kyung Hee University, Seoul, South Korea; ^3^Department of Neuroscience and Tissue Injury Defense Research Center, School of Medicine, Ewha Womans University, Seoul, South Korea; ^4^Ginsentology Research Laboratory and Department of Physiology, College of Veterinary Medicine and Bio/Molecular Informatics Center, Konkuk University, Seoul, South Korea; ^5^Institute of Korean Medicine, College of Korean Medicine, Kyung Hee University, Seoul, South Korea

**Keywords:** gintonin, 1-methyl-4-phenyl-1, 2, 3, 6-tetrahydropyridine, Parkinson’s disease, lysophosphatidic acid receptor, multi-target

## Abstract

Gintonin is a ginseng-derived lysophosphatidic acid receptor (LPAR) ligand. Although previous *in vitro* and *in vivo* studies demonstrated the therapeutic role of gintonin against Alzheimer’s disease, the neuroprotective effects of gintonin in Parkinson’s disease (PD) are still unknown. We investigated whether gintonin (50 and 100 mg/kg/day, p.o., daily for 12 days) had neuroprotective activities against neurotoxicity in a 1-methyl-4-phenyl-1,2,3,6-tetrahydropyridine (MPTP)-induced mouse model of PD. Pre-administration of 100 mg/kg gintonin displayed significantly ameliorating effects in neurological disorders (motor and welfare) as measuring using pole, rotarod, and nest building tests, and in the survival rate. These effects were associated to the reduction of the loss of tyrosine hydroxylase–positive neurons, microglial activation, activation of inflammatory mediators (interleukin-6, tumor necrosis factor, and cyclooxygenase-2), and alteration of blood-brain barrier (BBB) integrity in the substantia nigra pars compacta and/or striatum following MPTP injection. The benefits of gintonin treatment against MPTP also included the activation of the nuclear factor erythroid 2-related factor 2 pathways and the inhibition of phosphorylation of the mitogen-activated protein kinases and nuclear factor-kappa B signaling pathways. Interestingly, these neuroprotective effects of gintonin were blocked by LPAR1/3 antagonist, Ki16425. Overall, the present study shows that gintonin attenuates MPTP-induced neurotoxicity via multiple targets. Gintonin combats neuronal death, and acts as an anti-inflammatory and an anti-oxidant agent. It maintains BBB integrity. LPA receptors play a key role in gintonin-mediated anti-PD mechanisms. Finally, gintonin is a key agent for prevention and/or treatment of PD.

## Introduction

Parkinson’s disease (PD) is a common progressive neurodegenerative disorder characterized by profound loss of dopaminergic neurons and accumulation of α-synuclein aggregates into Lewy bodies and Lewy neuritis in the substantia nigra pars compacta (SNpc) of the midbrain, and by decreased dopamine levels in the striatum (caudate and putamen) of the basal ganglia (Shulman et al., 2011; Kalia and Lang, 2015; Poewe et al., 2017). The main symptoms of PD are movement impairments, such as slowness of movement, tremor, rigidity, and postural instability, and non-motor related disorders that include executive dysfunction, slowed cognitive speed, memory problems, genitourinary problems, and emotional changes (Shulman et al., 2011; Kalia and Lang, 2015; Poewe et al., 2017). PD has a complex, multi-factorial etiology including mitochondrial malfunction, glutamate excitotoxicity, apoptosis, oxidative stress, proteasomal dysfunction, and environmental exposures (Riess and Kruger, 1999; Shulman et al., 2011; Kalia and Lang, 2015; Poewe et al., 2017).

Although medicines for symptom relief including levodopa are the most trusted therapeutics for PD, the approach does not prevent the progressive loss of dopaminergic neurons, and does not result in cell regeneration in PD patients. In addition, they produce adverse effects including dizziness, nausea, and vomiting when therapy is prolonged (Marsden, 1994; Athauda and Foltynie, 2015). These unsatisfactory effects may be inextricably related to the targeting of only one of the multi-factorial mechanisms underlying neuronal degeneration (Riess and Kruger, 1999; Shulman et al., 2011; Kalia and Lang, 2015; Poewe et al., 2017). The recent and rapid advances in medical biology and technology have suggested a new paradigm of drug development (multi-targeted drugs) based on the multi-factorial and highly complex pathological features of various neurodegenerative disorders (Bajda et al., 2011; Dias and Viegas, 2014; Zheng et al., 2014; Calabresi and Di Filippo, 2015). The multi-targeted strategy may hopefully overcome the limitations of drugs directed at a single target. Plant-derived natural products that are proven safe, effective, and innovative, remain the best sources of drugs, encouraging continuous research, development and discovery of therapeutic approaches for a wide range of diseases and conditions (Newman and Cragg, 2012). Although increasing evidence suggests that characterizing and identifying potentially active natural products may meet the unmet demand of single-target drugs (Li et al., 2013), no approved PD protective medicines are currently available (Athauda and Foltynie, 2015).

*Panax (P.)* ginseng Meyer, a perennial herb of the family *Araliaceae*, has been widely used for millennia as an adaptogen, particularly in the eastern Asian countries including Korea, Chinese, and Japan (Cho, 2012; Lee et al., 2017). Beneficial effects of *P. ginseng* have been reported in various diseases including neurological disorders. The major active ingredients of *P. ginseng* are acidic polysaccharide, a carbohydrate polymer, and ginsenoside, a kind of plant saponin (Cho, 2012; Lee et al., 2017). Although the molecular mechanisms of the ingredients have been frequently studied in neurodegenerative diseases (Cho, 2012), they remain unclear. Recently, we isolated a novel ingredient from *P. ginseng*, termed gintonin. Gintonin is a non-carbohydrate polymer and a non-saponin (Choi et al., 2015b) that was identified as a novel ligand of G protein-coupled lysophosphatidic acid (Alpayci et al., 2012) receptors (LPARs). Gintonin elicits a transient increase in intracellular calcium concentration [Ca^2+^]_i_, which activates the calcium-dependent cellular events through the regulation of ion channels and cell surface receptors, induces anti-inflammatory activity by inhibiting mitogen-activated protein kinases (MAPKs) and nuclear factor-kappa B (NF-κB) pathways in lipopolysaccharide-induced RAW 264.7 cells, and increases the release of neurotransmitters (dopamine, catecholamine, and gliotransmitter) in cortical primary astrocytes and PC12 cells (Choi et al., 2015b; Saba et al., 2015). Intraperitoneal (i.p.) administration of gintonin to mice also increased serum dopamine concentrations (Hwang et al., 2015).

On the other hand, LPA receptors are present on most cell types and fluids within the developing and adult nervous system, and are functionally involved in many neural processes and pathways (Noguchi et al., 2009; Choi et al., 2010; Yung et al., 2014, 2015). Recent studies showed that dysregulations of brain LPA receptor pathways may lead to nervous system disorders including cognitive functions, hydrocephalus, neuropsychiatric disorders, and neuropathic pain (Noguchi et al., 2009; Choi et al., 2010; Yung et al., 2014, 2015), indicating that LPA receptors play important roles in maintenance of normal brain functions. In previous studies we demonstrated that gintonin exhibits *in vitro* and *in vivo* anti-Alzheimer’s disease, one of representative neurodegenerative diseases, effects via LPA receptor signaling pathway (Hwang et al., 2012). However, little is known on the effects of gintonin on PD. In the present study we investigated whether oral gintonin administration to MPTP-induced PD animal model attenuates brain neuropathies of PD and found that the gintonin administration exhibits multiple beneficial effects on PD via LPA receptors. Presently, we demonstrated that gintonin contributes to neuroprotections against MPTP-induced neurotoxicities in mice and further discuss possible molecular mechanisms on gintonin-mediated anti-PD in animal model.

## Materials and Methods

### Animals and Ethical Approval

Adult male C57BL/6 mice (Narabiotec Co., Ltd., Seoul, South Korea) that were 7–8 weeks of age and weighed 22–23 g) were housed at a constant temperature of 23 ± 2°C with a 12-h light-dark cycle (lights on from 08:00 to 20:00), and provided with food and water *ad libitum*. All experimental procedures were reviewed and approved by the Institutional Animal Care and Use Committee of Kyung Hee University [KHUASP(SE)-17-143]. In this process, proper randomization of laboratory animals and handling of data were performed in a blinded manner in accordance with recent recommendations from an NIH workshop on preclinical models of neurological diseases (Landis et al., 2012).

### Preparation of Gintonin and Its Composition

Gintonin was prepared as previous described (Choi et al., 2015a). Briefly, one kilogram of 4-year-old ginseng (Korea ginseng corporation, Daejeon, South Korea) was ground into small pieces (>3 mm) and refluxed with 70% fermented ethanol eight times for 8 h each at 80°C. The extracts (340 g) were concentrated, dissolved in distilled, cold water at a ratio of 1:10, and stored at 4°C for 24–96 h. The supernatant and precipitate fractions obtained by water fractionation after ethanol extraction of ginseng were separated by centrifugation (3,000 rpm, 20 min). The precipitate was lyophilized. Gintonin consists of carbohydrates, lipids and ginseng proteins. The proportion of total carbohydrates, lipids, and proteins in gintonin was approximately 30, 20.2, and 30.3%, respectively, in addition to other minor components (Choi et al., 2015a). The lipid composition of gintonin based on LC-MS/MS analysis is as follows: fatty acids (7.53% linoleic acid, 2.82% palmitic acid, and 1.46% oleic acid); lysophospholipids and phospholipids (0.60%); and phosphatidic acids (1.75%). The total lipid content in gintonin is about 14.2%. Qualitative assays indicate that gintonin also contains diacylglycerols and triacylglycerols (Choi et al., 2015a).

### Experimental Groups and Treatment With MPTP, Gintonin, and Ki16425

In order to determine the most effective dose and mechanism of pre-administration of gintonin, mice were randomly divided into sham, MPTP, MPTP + gintonin pre-administration (50 and 100 mg/kg), and gintonin groups. For MPTP injection, mice received four i.p. injections of MPTP-hydrochloride (20 mg/kg body weight; Sigma-Aldrich, St. Louis, MO, United States) dissolved in phosphate buffered saline (PBS) for 2 h intervals (Jackson-Lewis and Przedborski, 2007). Gintonin was dissolved in physiological saline and administrated orally at doses of 50 and 100 mg/kg once daily for 12 days from 5 days before the first MPTP injection. Ki16425, an antagonist against LPAR1 and LPAR3 (Tocris Bioscience, Bristol, United Kingdom), was prepared in 5% DMSO in PBS. It was administered once daily 30 min before gintonin treatment.

### Behavioral Assays

To examine motor coordination, mice (*n* = 5 per group) were subjected to pole and rotarod tests as previous described (Choi et al., 2018). The nest building behavior was measured as an indicator of health and welfare in mice as previous described (Choi et al., 2018). The behavioral tests were performed by an experimenter who was unaware of the experimental conditions and was done under constant temperature (23 ± 2°C) and humidity (55 ± 5%) in a quiet room, 1 day before and 1, 3, 5, and 7 days after MPTP injection.

### Immunohistochemical Evaluation

Seven days after the last injection of MPTP, brain (*n* = 5 per group) for histological evaluation were prepared as previously described (Jang et al., 2013; Lee et al., 2016). Sequential coronal sections (30 μm thickness) were acquired using a model CM3050S freezing microtome (Leica Biosystems, Wetzlar, Germany), from the level of the SNpc (bregma -2.54 to -3.40 mm) and mid-striatum (bregma +0.26 to +1.10 mm), according to the mouse brain atlas (Franklin and Paxinos, 2008). Immunohistochemical analysis of the SNpc and striatal sections was performed as previously described (Jang et al., 2013; Lee et al., 2016). Briefly, sections (*n* = 3 per brain) from all groups were incubated with either rabbit anti-tyrosine hydroxylase (TH; 1:1,000; Millipore, Bedford, MA, United States), rabbit anti-ionized calcium binding adapter molecule-1 (Iba-1; 1:2,000; WAKO, Osaka, Japan), rabbit anti-glial fibrillary acidic protein (GFAP; 1:5,000; DAKO, Carpinteria, CA, United States) or rat anti-platelet endothelial cell adhesion molecule-1 (PECAM-1; 1:500; Santa Cruz Biotechnology, Santa Cruz, CA, United States), followed by incubation with biotinylated rabbit IgG antibody (1:200; Vector Laboratories, Burlingame, CA, United States) and avidin–biotinylated horseradish peroxidase complex (1:200; Vector Laboratories). The sections were visualized with 3,3′-diamino-benzidine and cover-slipped with Permount.

### Western Blot Analysis

Seven days after the last injection of MPTP, the brains of all groups (*n* = 2–3 per group) was immediately removed with lysis buffer under anesthesia. Western blot analysis was accomplished as previously described (Jang et al., 2013; Lee et al., 2016). The polyvinylidene fluoride membranes with protein were probed overnight with rabbit anti-TH (1:1,000; Millipore), rabbit anti-Iba-1 (1:500; WAKO), mouse anti-GFAP (1:1,000; Millipore), rat anti-PECAM-1 (1:500; Santa Cruz Biotechnology), rabbit anti-phospho (p)-extracellular signal-regulated kinase (ERK), rabbit anti-ERK, rabbit anti-phospho (p)-c-Jun N-terminal kinase (JNK), rabbit anti-JNK, rabbit anti-p-p38, rabbit anti-p38, rabbit anti-p-NF-κB p65, rabbit anti-NF-κB p65, rabbit anti-p-IκBα, mouse anti-IκBα (1:1,000; Cell Signaling Technology, Beverly, MA, United States), rabbit anti-nuclear factor erythroid 2-related factor 2 (Nrf2; 1:1000, Santa Cruz Biotechnology), mouse anti-heme oxygenase-1 (HO-1; 1:1,000; Enzo Life Sciences, Farmingdale, NY, United States), mouse anti-NQO1 (1:1,000; Cell Signaling Technology), rabbit anti-LPAR1 (1:1,000; Abcam, Cambridge, United Kingdom), or rabbit anti-LPAR3 (1:1,000; Abcam) at 4°C, followed by incubation with horseradish peroxidase-conjugated secondary antibody for 1 h prior to enhanced chemiluminescence analysis (Amersham Pharmacia Biotech, Piscataway, NJ, United States) and visualized using a super cooled-CCD camera system with a Davinch-K Gel imaging system (Dvinch-K, Seoul, South Korea). For normalization of the antibody signal, the membranes were stripped and reprobed with glyceraldehyde 3-phosphate dehydrogenase (GAPDH; 1:5,000; Cell Signaling Technology) or total antibody levels against each protein. After Western blot was performed three times, the density of each band was converted to a numerical value using the Photoshop CS2 program (Adobe, San Jose, CA, United States) after subtracting background values from an area of film immediately adjacent to the stained band. Data are expressed as the ratio of each expression against GAPDH or total protein in each sample. Original images of Western blots were supported in **Supplementary Figure S2**.

### Real-Time Polymerase Chain Reaction (PCR) Analysis

Seven days after the MPTP injection, brain of each mouse (*n* = 3 per group) was rapidly removed under anesthesia, coronal brain slices (3 mm thickness) were prepared on ice-cold subbed slide glass using a brain matrix device (Roboz Surgical Instrument Co. Gaithersburg, MD, United States), and SNpc and striatum regions were sampled using microscissors and blade under a dissection microscope. Real-time PCR analysis was accomplished as previously described (Choi et al., 2016). The mRNA levels of each target gene were normalized to that of GAPDH mRNA. Fold-induction was calculated using the 2^-ΔΔC_T_^ method as previously described (Livak and Schmittgen, 2001). The primer sequences were as follows; interleukin (IL)-6-5′-TCC ATC CAG TTG CCT TCT TGG-3′ and 5′-CCA CGA TTT CCC AGA GAA CAT G-3′, tumor necrosis factor (TNF)-α-5′-AGC AAA CCA CCA AGT GGA GGA-3′ and 5′-GCT GGC ACC ACT AGT TGG TTG T-3′, Cyclooxygenase (COX)-2-5′-CAG TAT CAG AAC CGC ATT GCC-3′ and 5′-GAG CAA GTC CGT GTT CAA GGA-3′, endothelial intercellular adhesion molecule (ICAM)-1-5′-TGC GTT TTG GAG CTA GCG GAC CA-3′ and 5′-CGA GGA CCA TAC AGC ACG TGC AG-3′, vascular cell adhesion molecule (VCAM)-1-5′-CCT CAC TTG CAG CAC TAC GGG CT-3′ and 5′-TTT TCC AAT ATC CTC AAT GAC GGG-3′, zoula occludens (ZO)-1-5′-AAG GCA ATT CCG TAT CGT TG-3′ and 5′-CCA CAG CTG AAG GAC TCA CA-3′, claudin-3-5′-CTG GGA GGG CCT GTG GAT GAA CT-3′ and 5′-TCG CGG CGC AGA ATA GAG GAT-3′, LPAR1-5′-GAG GAA TCG GGA CAC CAT GAT-3′ and 5′-ACA TCC AGC AAT AAC AAG ACC AAT C-3′, LPAR2-5′-GAC CAC ACT CAG CCT AGT CAA GAC-3′ and 5′-CTT ACA GTC CAG GCC ATC CA-3′, LPAR3-5′-GCT CCC ATG AAG CTA ATG AAG ACA-3′ and 5′-AGG CCG TCC AGC AGC AGA-3′, LPAR4-5′-CAG TGC CTC CCT GTT TGT CTT C-3′ and 5′-GAG AGG GCC AGG TTG GTG AT-3′, LPAR5-5′-GCT CCA GTG CCC TGA CTA TC-3′ and 5′-GGG AAG TGA CAG GGT GAA GA-3′, LPAR6-5′-ACA GTG ATG GGA GGA AGT GC-3′ and 5′-CCG CTG GAA AGT TCT CAA AG-3′, and GAPDH-5′-AGG TCA TCC CAG AGC TGA ACG-3′ and 5′-CAC CCT GTT GCT GTA GCC GTA T-3′.

### Statistical Analyses

All data are presented as means ± SEM. Statistical analyses were performed using the SPSS 23.0 package (SPSS Inc, Chicago, IL, United States) for Windows. Two-sample comparisons were carried out using the Student’s *t*-test and multiple comparisons were made using two-way ANOVA with Tukey’s *post hoc* test. Statistical difference was identified at the 5% level unless otherwise indicated.

## Results

### Gintonin Improves Neurological Impairments and Dopaminergic Neuronal Death Following MPTP Injection

**FIGURE 1:**
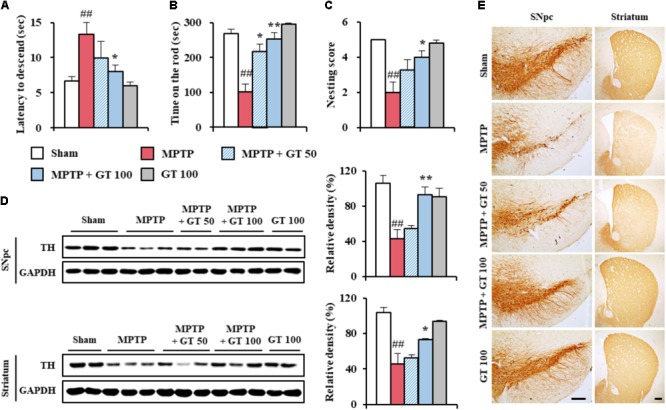
Gintonin attenuates neurological impairment and dopaminergic cell death by MPTP neurotoxicity. **(A–C)** Mice (*n* = 5 per group) were orally administrated gintonin (50 and 100 mg/kg/day) or saline from 1 h before the first MPTP injection (20 mg/kg, every 2 h × 4 times). Pole test **(A)**, rotarod performance test **(B)**, and nest building behavior test **(C)** were performed 3, 5, and 1 days after the last MPTP-injection. **(D)** SNpc and striatum each group (*n* = 2–3 per group) were sampled 7 days after MPTP injection and quantitatively analyzed by immunoblot analysis using TH antiserum. The left panels illustrate representative Western blots. **(E)** SNpc and striatum sections (*n* = 3 per brain) from each group (*n* = 5 per group) were prepared 7 days after MPTP injection and immunostained with TH antiserum. Scale bar = 100 μm. ANOVA test; ^##^*p* < 0.01 vs. Sham group; ^∗^*p* < 0.05 and ^∗∗^*p* < 0.01 vs. MPTP group.

To determine the effective dose of gintonin for the treatment of MPTP-mediated neurological impairment, we investigated the motor coordination in mice. First, we performed pole test. In the MPTP group, the average descent time from the top to the bottom of the pole increased by 98.5% (13.3 ± 1.7 s) compared with the sham group (6.7 ± 0.6 s). The average descent time was decreased by 24.8–39.8% by gintonin administration (10.0 ± 2.3 and 8.0 ± 0.9 s for 50 and 100 mg/kg/day gintonin, respectively; **Figure 1A**). One hour after the pole test, rotarod performance was tested. In the MPTP group, the average latency to fall decreased by 62.0% (101.6 ± 21.7 s) compared with the sham group (267.6 ± 12.6 s), while the average latency to fall was increased by 114.5–149.0% by gintonin administration (218.0 ± 19.7 and 253.0 ± 17.9 s for 50 and 100 mg/kg/day gintonin, respectively; **Figure 1B**). As an indicator of health and welfare, nest building behavior was measured. In the MPTP group, the mean score of the quality of the resulting nest decreased by 60% (2.0 ± 0.6) compared with the sham group (5.0 ± 0.0). However, the mean score was improved by 100% following gintonin administration (4.0 ± 0.4 in the 100 mg/kg/day gintonin) compared to the MPTP group (**Figure 1C**). At the end of the experiment, the survival rate of all groups was 100% (data not shown).

Since MPTP-mediated neurological disorders results from the loss of dopaminergic neurons in the SNpc and depletion of the dopamine in the striatum (Gubellini and Kachidian, 2015), we investigated whether gintonin could prevent the loss of dopaminergic neurons/fibers by immunoblotting and immunohistochemical analyses using TH antibody 7 days after MPTP injection (**Figures 1D,E**). The expression of TH protein in the SNpc was reduced by MPTP-injection (42.7 ± 6.0%) compared to the sham group (105.7 ± 6.0%), while the reduction was significantly inhibited by gintonin administration (54.9 ± 3.8% and 92.9 ± 6.4% for 50 and 100 mg/kg/day gintonin, respectively; upper panel of **Figure 1D**). The findings agreed with the alteration in intensity of TH immunoreactivity (**Figure 1E**). Since the fibers of dopaminergic neurons in the SNpc project to the striatum (Gubellini and Kachidian, 2015; Kalinderi et al., 2016), we examined the alteration of TH immunoreactivity in the striatum. As expected, the reduction of striatal TH protein expressions by MPTP neurotoxicity (45.5 ± 3.4%) were also inhibited by gintonin (52.7 ± 3.9% and 72.8 ± 12.3% for 50 and 100 mg/kg/day gintonin, respectively; lower panel of **Figure 1D**), in agreement with the alteration of intensity of TH immunoreactiviy (**Figure 1E**). Gintonin did not induce significant alteration in TH expression in the SNpc and striatum. The findings suggest that gintonin may inhibit MPTP-mediated neurological impairments by decreasing dopaminergic degeneration in the SNpc and striatum.

### Gintonin Inhibits Microglial Activation and the Expression of Inflammatory Mediators in the SNpc or Striatum Following MPTP Injection

**FIGURE 2:**
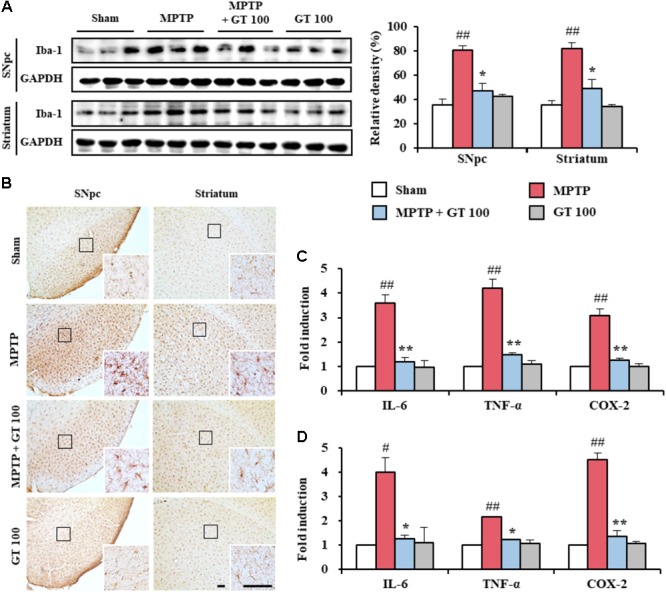
Gintonin attenuates activation of microglia and inflammatory mediators in the SNpc and striatum after MPTP injection. **(A)** SNpc and striatum from each group (*n* = 3 per group) 7 days after MPTP-injection were quantified by immunoblot analysis using Iba-1 antiserum. The left panels illustrate representative Western blots. **(B)** SNpc and striatum sections (*n* = 3 per brain) 7 days after MPTP injection were immunostained with Iba-1 antiserum. Insets display high magnification micrographs of the areas marked with squares. **(C,D)** Real-time PCR was used to quantify mRNA expression of IL-6, TNF-α, and COX-2 in SNpc and striatum (*n* = 3 per group) from day 7 after MPTP injection (SNpc, **C**; striatum, **D**). Scale bar = 50 μm. ANOVA test; ^#^*p* < 0.05 and ^##^*p* < 0.01 vs. Sham group; ^∗^*p* < 0.05 and ^∗∗^*p* < 0.01 vs. MPTP group.

Since microglia are activated within or around lesions of neurodegenerative disorders, such as PD, and activated microglia contribute to neurodegeneration by the producing inflammatory mediators (Liberatore et al., 1999; Lobsiger and Cleveland, 2007; Du et al., 2017), we wondered whether gintonin could have neuroprotective effects closely related with the down-regulation of the anti-inflammatory response. The level of Iba-1 (a marker for microglia) protein expression was enhanced in the SNpc (80.8 ± 3.2%) and striatum (81.5 ± 5.3%) from MPTP group compared to the sham group (35.6 ± 4.6% in the SNpc and 35.2 ± 3.7% in the striatum), whereas the enhancement was inhibited by 100 mg/kg of gintonin administration (47.0 ± 6.5% in the SNpc and 49.2 ± 7.3% in the striatum) compared to the MPTP group (**Figure 2A**). The expression trend paralleled the alteration in the intensity of Iba-1-immunoreactivity (**Figure 2B**). In the SNpc and striatum from the MPTP group, Iba-1-immunoreactive cells showed typically activated form with enlarged cell bodies and short and thick processes compared to the sham group, which generally displayed the typical forms of resting cells that included small cell bodies and thin processes (Jang et al., 2013; Jang and Cho, 2016; Lee et al., 2016). However, the morphology of Iba-1-immunoreactive cells from gintonin-administrated group was relatively similar to that of the resting cells from the sham group.

Since activated microglia may produce inflammatory mediators, which have been implicated in the degeneration of dopaminergic neurons in the SNpc and striatum from MPTP model of PD (Liberatore et al., 1999; Lobsiger and Cleveland, 2007; Du et al., 2017), we investigated whether gintonin might down-regulate the representative inflammatory mediators in the SNpc and striatum after MPTP injection. Real-time PCR analysis demonstrated that the relative mRNA expressions of IL-6, TNF-α, and COX-2 were enhanced by 3.6-, 4.2-, and 3.1-fold, respectively, in the SNpc, and by 4.0-, 2.1-, and 4.5-fold, respectively, in the striatum, 7 days following MPTP injection compared to sham group. In contrast, their enhancements were significantly prevented in the SNpc (66.7, 64.3, and 61.3%, respectively), and in the striatum (67.5, 42.9, and 71.1%, respectively) following the administration of 100 mg/kg gintonin compared with the MPTP group. Real-time PCR analysis revealed little or no mRNA expression of both genes in the SNpc and striatum from the sham groups (**Figures 2C,D**). The findings suggest that gintonin might contribute to neuroprotection against MPTP-mediated neurotoxicity by inhibiting microglial activation and the inflammatory response.

### Gintonin Blocks MAPKs and NF-κB Pathways in the Striatum Following MPTP Injection

**FIGURE 3:**
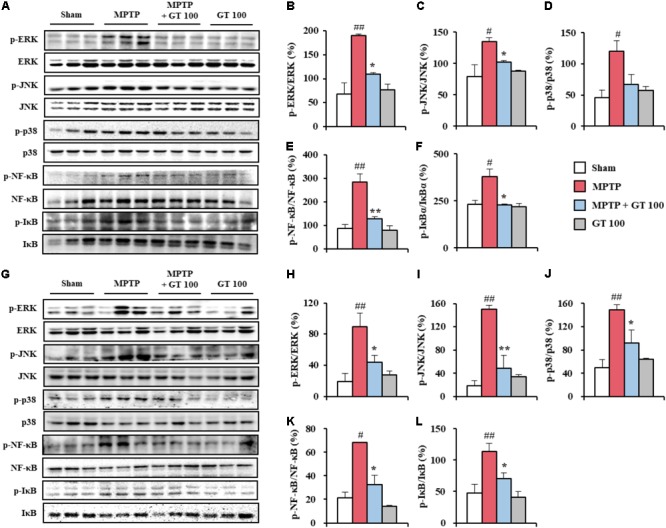
Gintonin inhibits MAPKs and NF-κB signaling pathways in the SNpc and striatum after MPTP injection. **(A–L)** SNpc and striatum sample from each group (*n* = 3 per group) 7 days after MPTP-injection were quantified by immunoblot analysis to measure the alteration in the MAPKs **(A–D,G–J)** and NF-κB pathways **(A,E,F,G,K,L)**. SNpc **(A–F)** and striatum **(G–L)**. The left panels illustrate representative Western blots **(A,G)**. ANOVA test; ^#^*p* < 0.05 and ^##^*p* < 0.01 vs. Sham group. ^∗^*p* < 0.05 and ^∗∗^*p* < 0.01 vs. MPTP group.

Since MAPK and NF-κB pathways have been implicated as a main signaling pathway in the neuronal death, oxidative stress, and BBB disruption (Abbott et al., 2006; Sandoval and Witt, 2008; Zlokovic, 2008), we measured the regulation effect of gintonin on the both pathways in the SNpc and striatum after MPTP injection. The activation of ERK, JNK, and p38 proteins was significantly enhanced in the SNpc (189.8 ± 3.3%, 134.9 ± 5.9%, and 120.0 ± 17.2%, respectively) and striatum (89.5 ± 17.6%, 150.6 ± 6.7%, and 148.7 ± 9.0%, respectively) 7 days after MPTP injection compared to the sham group (68.4 ± 23.3%, 79.0 ± 18.4%, and 45.7 ± 12.5%, respectively, in the SNpc, and 19.1 ± 10.2%, 19.0 ± 8.7%, and 49.4 ± 14.0%, respectively). The enhancement of activation was blocked by pretreatment with 100 mg/kg gintonin (108.9 ± 4.1%, 102.2 ± 2.3%, and 67.5 ± 15.3%, respectively, in the SNpc, and 43.7 ± 9.0%, 49.2 ± 22.2%, and 92.6 ± 21.8%, respectively, in striatum) (**Figures 3A–D,G–J**). Subsequently, we examined whether gintonin regulates the NF-κB pathway in the SNpc and striatum following MPTP injection. Expressions of p-NF-κB and p-IκBα were significantly increased in the SNpc (283.4 ± 36.6%, and 379.6 ± 40.7%, respectively) and striatum (68.4 ± 0.1% and 114.0 ± 12.8%, respectively) 7 days after the MPTP-injection compared to the sham group (87.2 ± 17.1%, and 231.9 ± 21.1%, respectively, in the SNpc, and 21.4 ± 4.5% and 47.3 ± 13.9%, respectively, in the striatum). The expressions were significantly increased by the administration of 100 mg/kg gintonin (128.5 ± 8.7%, and 227.5 ± 5.5%, respectively, in the SNpc, and 32.5 ± 7.9% and 71.0 ± 8.5%, respectively, in the striatum) (**Figures 3A,E,F,G,K,L**). Phosphorylation of MAPKs or NF-κB pathways was not significantly affected by gintonin administration alone (**Figures 3A–L**). The results suggest that gintonin might diminish MPTP neurotoxicity by inhibiting the activation of the MAPKs and NF-κB pathways.

### Gintonin Activates Nrf2 Pathway in the Striatum Following MPTP Injection

**FIGURE 4:**
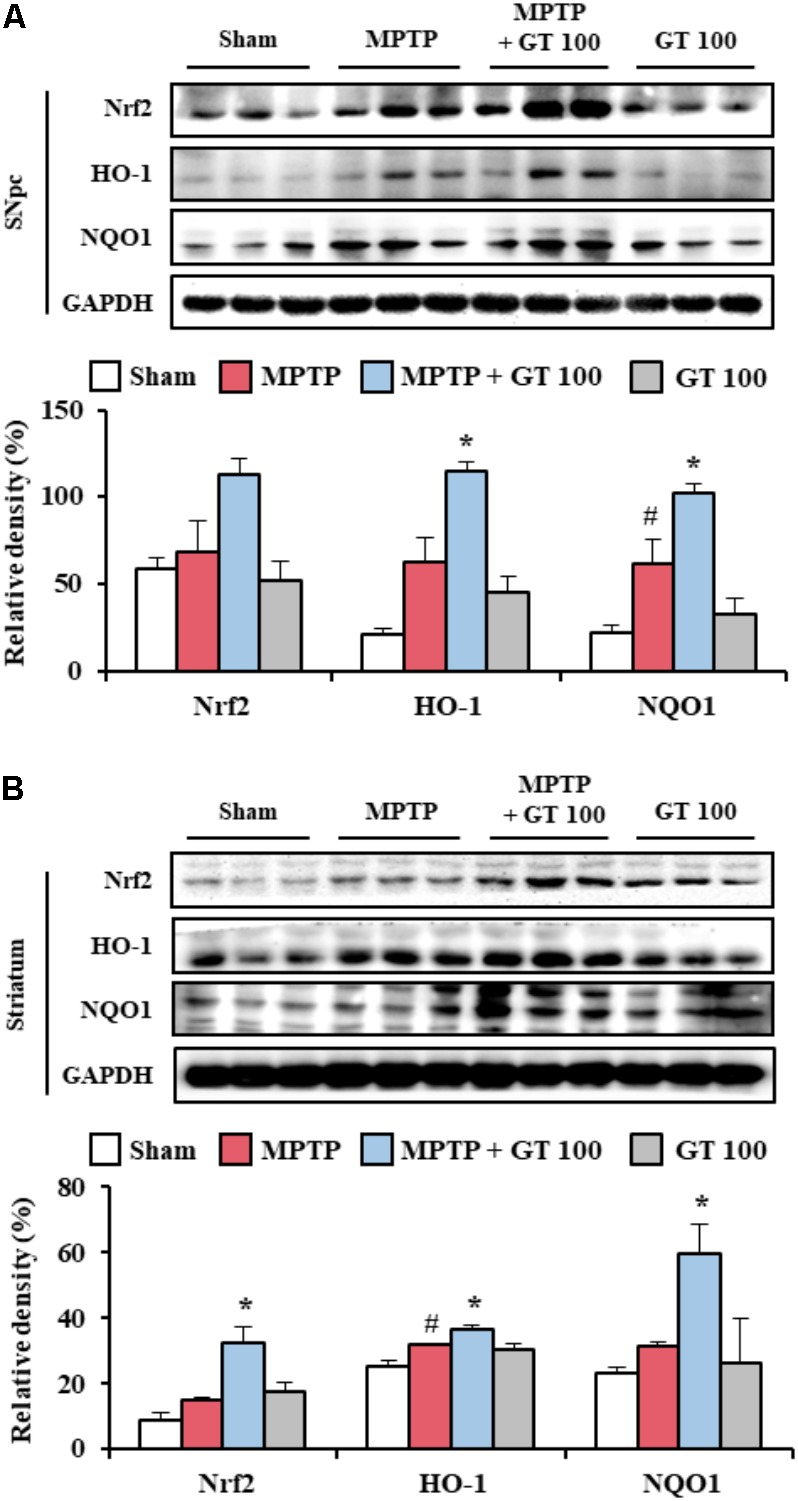
Gintonin activates Nrf2 signaling pathway in the SNpc and striatum after MPTP injection. **(A,B)** SNpc and striatum sample (*n* = 3 per group) 7 days after MPTP-injection were quantified by immunoblot analysis. Nrf2, HO-1, and NQO1. SNpc **(A)** and striatum **(B)**. The top panels illustrate representative Western blots. ANOVA test; ^#^*p* < 0.05 vs. Sham group. ^∗^*p* < 0.05 vs. MPTP group.

*Panax ginseng* extract and ginsenosides exert anti-oxidative effects through Nrf2 transcriptional activation in neural dysfunctions (Nabavi et al., 2015; Lee et al., 2017). Yet, the anti-oxidative effects of See comment in PubMed Commons below gintonin are unknown. Therefore, we examined the effect of gintonin on the Nrf2 pathway in the MPTP-mediated PD model by immunoblot blot analysis. The level of Nrf2 protein expression was slightly increased in the SNpc (68.1 ± 18.1%) and striatum (14.7 ± 0.7%) by MPTP injection compared to the sham group (59.4 ± 6.0% in the SNpc and 8.9 ± 2.0% in the striatum), while they further increased by administration with 100 mg/kg of gintonin (113.2 ± 9.0% in the SNpc and 32.1 ± 5.1% in the striatum) (**Figures 4A,B**). Consequently, the expression of the phase II enzymes heme oxidase-1 (HO-1), and NAD(P)H:quinone oxidoreductase 1 (NQO-1) was increased by 62.3 ± 13.7% and 61.7 ± 11.4%, respectively, in the SNpc, and by 31.7 ± 0.6% and 31.4 ± 1.1%, respectively, in striatum of the gintonin-administrated group compared to that of MPTP group (114.2 ± 5.4% and 101.7 ± 5.7%, respectively, in the SNpc, and 36.7 ± 0.6% and 59.6 ± 9.2%, respectively, in striatum) (**Figures 4A,B**). The results suggest that antioxidant effect of gintonin may contribute to its neuroprotective effects in the MPTP-mediated neurotoxicity.

### Gintonin Protects BBB Integrity After MPTP Injection

**FIGURE 5:**
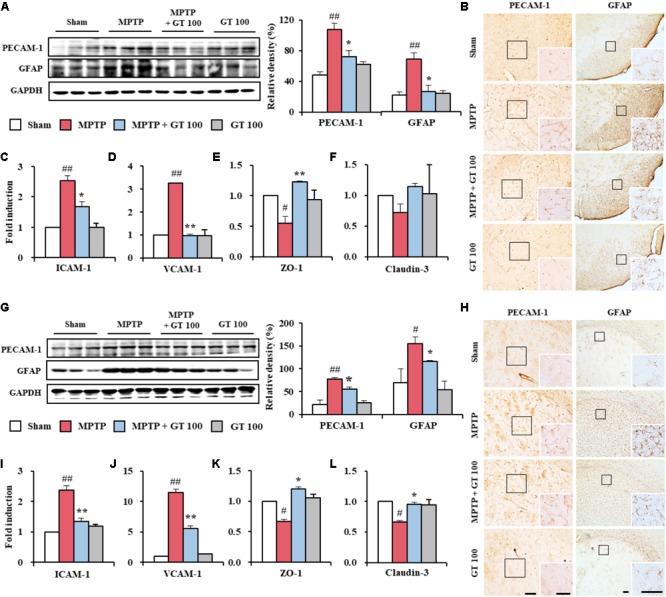
Gintonin prevents disruption of the BBB integrity in the SNpc and striatum after MPTP injection. **(A,G)** SNpc and striatum sample (*n* = 3 per group) were quantified by immunoblot analysis using PECAM-1 and GFAP antisera. The left panels illustrate representative Western blots. **(B,H)** SNpc and striatum sections (*n* = 3 per brain) from each group (*n* = 5 per group) 7 days after MPTP-injection were immunostained using PECAM-1 and GFAP antisera. Insets display high magnification micrographs of the areas marked with squares. Scale bar = 50 μm. **(C–F,I–L)** Real-time PCR using primers for ICAM-1 **(C,I)**, VCAM-1 **(D,J)**, ZO-1 **(E,K)**, and claudin-3 **(F,L)** was used to quantify the expression of these molecules in SNpc **(C–F)** and striatum **(I–L)** (*n* = 3 per group) 7 days after MPTP-injection. ANOVA test; ^#^*p* < 0.05 and ^##^*p* < 0.01 vs. Sham group. ^∗^*p* < 0.05 and ^∗∗^*p* < 0.01 vs. MPTP group.

Since the BBB was disrupted during neurological disorders including PD (Abbott et al., 2006; Zlokovic, 2008), we examined the effect of gintonin on the level of BBB disruption and maintenance of BBB integrity 7 days after MPTP injection. Expression of PECAM-1, a marker of BBB disruption, was increased in the SNpc (107.3 ± 10.2%) and striatum (77.5 ± 3.1%) of the MPTP group compared to sham group (48.3 ± 0.9% in the SNpc and 21.2 ± 9.9% in the striatum). The increase was inhibited by the administration of 100 mg/kg gintonin (72.0 ± 4.7% and 55.4 ± 3.9%, respectively) (**Figures 5A,G**), in accordance with the alteration of PECAM-1 immunoreactivity (**Figures 5B,H**). Intensity of expression of GFAP protein, one of the main components of BBB (Abbott et al., 2006), was markedly increased in the SNpc (69.7 ± 8.1%) and striatum (155.9 ± 14.4%) of the MPTP group compared to sham group (22.4 ± 3.9% in the SNpc and 69.8 ± 31.1% in striatum). The increase was significantly inhibited by gintonin (26.6 ± 8.4% in the SNpc and 116.8 ± 1.9% in striatum) (**Figures 5A,G**), in accordance with the alteration of GFAP-immunoreactivity (**Figures 5B,H**). We tested the effect of gintonin on the changes of adhesion and junctional molecules. Real-time PCR analysis revealed increased mRNA expression of ICAM-1 and VCAM-1, representative adhesion molecules, in the SNpc (2.5 and 3.2-fold, respectively) and striatum (2.4 and 11.5-fold, respectively) following MPTP injection compared to the sham group, whereas the increase was blocked by the gintonin administration (32.0 and 68.8%, respectively, in the SNpc and 45.8 and 52.2%, respectively, in the striatum) (**Figures 5C,D,I,J**). Meanwhile, mRNA expressions of ZO-1 and claudin-3, representative junctional molecules, were reduced in the SNpc (0.6 and 0.7-fold, respectively) and striatum (0.7 and 0.7-fold, respectively) following MPTP injection. The reduction was inhibited by gintonin (100.0 and 42.9%, respectively, in the SNpc and 71.4 and 42.6%, respectively, in the striatum) (**Figures 5E,F,K,L**). The collective data demonstrate that the positive activity of gintonin on the disruption and maintenance of BBB might contribute to its protective effect against MPTP toxicity.

### Gintonin Activates LPARs Pathways in the SNpc and Striatum After MPTP Injection

**FIGURE 6:**
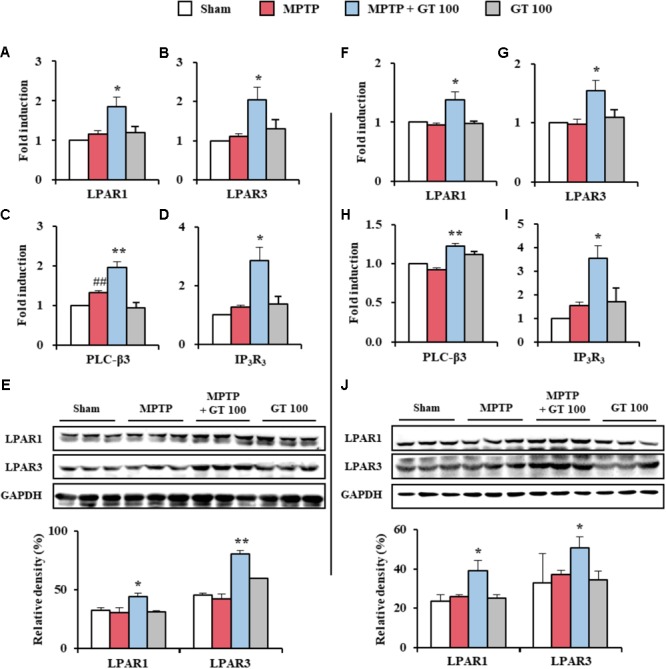
Gintonin activates LPAR1 and 3 signaling pathways in the SNpc and striatum after MPTP injection. **(A–J)** SNpc and striatum sample from each group (*n* = 3 per group) 7 days after MPTP-injection were quantified by real-time PCR **(A–D,F–I)** and immunoblot analysis **(E,J)** to measure the alteration in expression of LPAR 1 and 3 or their downstream molecules. mRNA expression of LPAR1 **(A,F)**, LPAR3 **(B,G)**, PLC-β3 **(C,H)**, and IP_3_R_3_ **(D,I)**. Protein expression of LPAR1 and 3 **(E,J)**. The top panels illustrate representative Western blots **(E,J)**. SNpc **(A–E)** and striatum **(F–J)**. ANOVA test; ^##^*p* < 0.01 vs. Sham group. ^∗^*p* < 0.05 and ^∗∗^p < 0.01 vs. MPTP group.

Since gintonin as an exogenous LPA induces various cellular effects that include migration and cell proliferation through the activation of LPARs (Choi et al., 2015b), we tested the expression pattern of LPARs. Interestingly, mRNA expressions of LPAR 1 and 3 were slightly (but not significantly) increased in the SNpc and striatum by MPTP-mediated neurotoxicity. The expressions were further increased by administration of 100 mg/kg gintonin (**Figures 6A,B,F,G**), in agreement with the alteration in the mRNA expression of phospholipase C-β3 and IP_3_R_3_, which are representative molecules in the downstream cascade (**Figures 6C,D,H,I**) and protein expression of LPAR 1 and 3 (**Figures 6E,J**). However, mRNA expression of other types of LPAR was not significantly affected by MPTP or gintonin (**Supplementary Figure S1**). The overall results suggest a possible role of LPAR1 and LPAR3 in gintonin-mediated anti-PD effects in brain.

### Multi-Target Effects of Gintonin Are Neutralized by Ki16425 Treatment

**FIGURE 7:**
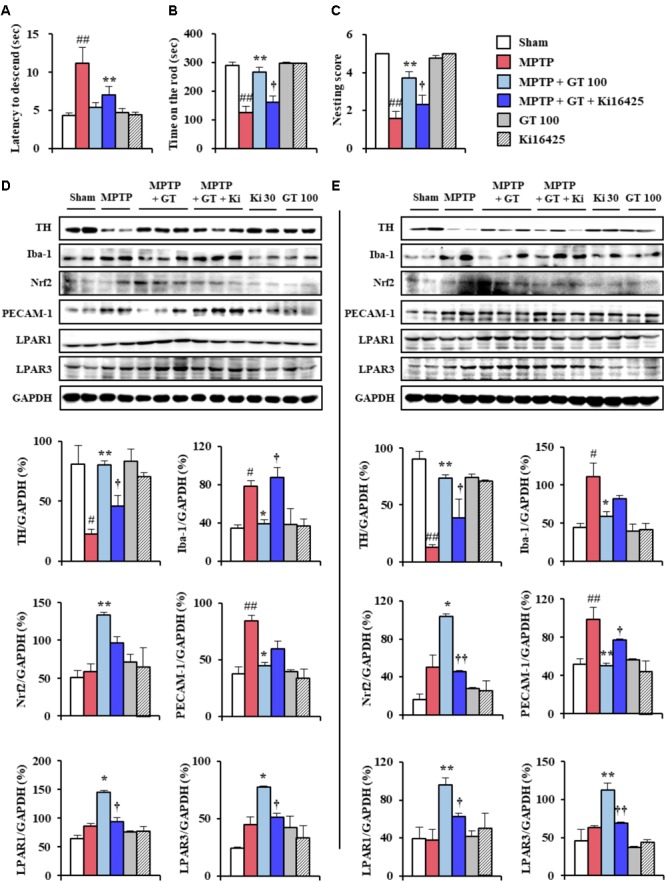
Multi-target effects of gintonin in MPTP-induced neurotoxicity were neutralized by Ki16425, an LPAR1/3 antagonist. **(A–C)** Mice (*n* = 8 per group) were orally administered with gintonin or saline 1 h before the first MPTP injection. Ki16425 was used once daily 30 min before gintonin treatment. Pole test **(A)**, rotarod performance test **(B)**, and nest-building behavior test **(C)** were conducted 3, 3, and 1 days after the last MPTP-injection. **(D,E)** Immunoblot analysis of TH, Iba-1, Nrf2, PECAM-1, LPAR1, and LPAR3 was conducted in SNpc **(D)** and striatum **(E)** obtained from each group (*n* = 2–3 per group) 7 days after MPTP injection. The top panels illustrate representative Western blots. ANOVA test; ^#^*p* < 0.05 and ^##^*p* < 0.01 vs. Sham group. ^∗^*p* < 0.05 and ^∗∗^*p* < 0.01 vs. MPTP group. ^†^*p* < 0.05 and ^††^*p* < 0.01 vs. MPTP + GT 100 mg/kg/day group.

Gintonin significantly enhanced the expression of LPAR1 and 3 (**Figure 6**), resulting in multi-target effects. Gintonin combats neuronal death, exerts anti-inflammatory and anti-oxidant effects, and contributes to the maintenance of BBB integrity in the SNpc and striatum after MPTP injection (**Figures 1–6**). The results strongly suggest the possibility that interruption of the LPA pathway neutralizes the effects of gintonin on MPTP neurotoxicity. To investigate this possibility, we intraperitoneally administered Ki16425 (a LPAR1 and 3 antagonist) to mice once daily 30 min before gintonin treatment in an MPTP model. As expected, the protective effects of gintonin against neurological disorders in pole, rotarod, and nest-building tests after MPTP injection was significantly neutralized by Ki16425 (**Figures 7A–C**). Further, the increased expression of TH protein by gintonin was neutralized by Ki16425 (**Figures 7D,E**), consistent with the neutralization of gintonin expression in Iba-1, Nrf2, and PECAM-1 expression (**Figures 7D,E**). Conclusively, enhanced protein expression of LAPR1 and LPAR3 after gintonin treatment was blocked by Ki16425 (**Figures 7D,E**). The results indicate that the beneficial effects of gintonin after MPTP injection were neutralized by interrupting LPA signaling before treatment.

## Discussion

The pathogenesis underlying the loss of dopaminergic neurons in PD remains subject to further debate, although the cell death is multifactorial and to be associated with mitochondrial malfunction, apoptosis, oxidative stress, and inflammation (Riess and Kruger, 1999; Shulman et al., 2011; Blesa et al., 2015; Kalia and Lang, 2015; Poewe et al., 2017). Currently, most single-target therapeutics, such as levodopa, provide symptomatic relief with adverse effects when PD patients receive long-term therapy (Marsden, 1994; Guneysel et al., 2008). Therefore, to overcome these limitations, the desire to develop more effective and safer therapeutic approaches for PD is driving drug design toward multi-target compounds acting in the central nervous system designed from natural products (Bajda et al., 2011; Dias and Viegas, 2014; Zheng et al., 2014; Calabresi and Di Filippo, 2015). Gintonin is a ligand of LPARs that was isolated from *P. ginseng*, a recognized well-known medicinal herb that has been widely used in traditional medicine to treat various diseases, including motor disabilities (Nah, 2012; Choi et al., 2015b; Hwang et al., 2015; Kim et al., 2017). The present data demonstrate the protective effects of gintonin in the MPTP-mediated SNpc and striatal toxicity through multifunctional activities including anti-neuronal death, anti-inflammation, anti-oxidant, and inhibition of BBB disruption. Thus, gintonin has potential value in functional foods and new drugs to preventive and treat PD, based on its multi-target effects.

Neuroinflammation and neuroimmune dysfunction might be closely related with the chronic features of neurodegenerative diseases, such as PD (Lobsiger and Cleveland, 2007; Du et al., 2017). Microglia are important in the development and maintenance of the brain micro-environment during inflammatory response. Activated microglia are pivotal cells in the defense against immunopathogenesis of infections and neurodegenerative disorders (Lobsiger and Cleveland, 2007; Du et al., 2017). Therefore, controlling microglial activation is considered as an attractive trial to protect dopaminergic neurons in the *in vivo* model of PD and PD patients (Du et al., 2017). *P. ginseng* extract has anti-inflammatory role in various *in vitro* and *in vivo* studies (Cho, 2012; Choi et al., 2015b; Nabavi et al., 2015; Lee et al., 2017), and gintonin suppresses the increase in the expression of pro-inflammatory cytokines (IL-1β, IL-6, and TNF-α), COX-2, and iNOS in lipopolysaccharide-stimulated RAW 264.7 cells (Saba et al., 2015). In accordance with these reports, gintonin suppressed microglial activation and up-regulation of mRNA expressions of pro-inflammatory cytokines (IL-6, and TNF-α), and COX-2 in the SNpc and striatum following MPTP injection (**Figure 2**). Collectively, our results indicate that gintonin can exert suppressive effect dopaminergic neurodegeneration by MPTP neurotoxicity by attenuating microglial activation and its inflammatory responses.

The pathways of MAPKs and NF-κB regulate the expression of many genes involved in a variety of processes including neurodegeneration, neuroinflammation, oxidative stress, and BBB disruption (Abbott et al., 2006; Sandoval and Witt, 2008; Zlokovic, 2008). Agents to control MAPKs and NF-κB pathways may be potential medications with an ability to prevent or treat PD through various mechanisms (Kim and Choi, 2010; Flood et al., 2011). In present study, as expected, administration of gintonin inhibited phosphorylation of all three MAPKs as well as phosphorylation of NF-κB and IκBα in the SNpc and striatum following MPTP injection (**Figure 3**). Previous studies have demonstrated that gintonin inhibits inflammation by MAPKs (p-ERK, p-JNK, and p-p38) and NF-κB pathways (NF-κB and p-IκBα) in lipopolysaccharide-induced RAW 264.7 cells (Saba et al., 2015). The observations along with the present findings that gintonin activates Nrf2 and Nrf2-dependent genes and their proteins including HO-1 and NQO-1 (**Figure 4**), supports the hypothesis that triggering the Nrf2 pathway by gintonin may block activation of MAPKs and NF-κB pathways, contributing to gintonin’s anti-inflammatory activity against MPTP neurotoxicity. Collectively, our findings indicate that gintonin can mitigate dopaminergic cell death by MPTP toxicity via anti-inflammatory activity by inhibiting the MAPKs and NF-κB mediated pathways by the triggered Nrf2 pathway.

The processes of oxidative stress induced by reactive oxygen species are a cause of a complex multifactorial PD (Riess and Kruger, 1999; Blesa et al., 2015). Recently, Nrf2, a phase II antioxidant ‘master regulator,’ was reported to demonstrably mitigate the neurotoxic actions of parkinsonian agents such as MPP^+^, rotenone, and hydrogen peroxide *in vitro* and *in vivo* (Todorovic et al., 2016). Many natural products including sulforaphane, which is present in broccoli, and ginsenosides of *P. ginseng* protects neuronal cells by activating Nrf2-mediated signaling *in vitro* and *in vivo* (Cho, 2012; Nabavi et al., 2015; Jang and Cho, 2016). It is conceivable, therefore, that the natural product-derived pharmacological modulators of the Nrf2 pathway may be very beneficial against neural toxicity. In present study, gintonin activated Nrf2 and representative Nrf2-dependent proteins, HO-1 and NQO1, in the SNpc and striatum following MPTP injection (**Figure 4**). The results may be supported by anti-oxidant effect of *P. ginseng* extract, fractions, and ginsenosides by activation of Nrf2 pathway *in vitro* and *in vivo* (Ong et al., 2015). Taken together, the data indicate that gintonin contributes to anti-dopaminergic cell death via anti-oxidant activity.

The blood-brain barrier (BBB) is a multicellular vascular structure that consists of the foot processes of astrocytes, pericytes, and endothelial cells. Inter-endothelial connections contain a variety of junctional molecule species, such as adherens, tight, and gap junctions (Abbott et al., 2006; Zlokovic, 2008). The BBB restricts the passage of various biological or chemical entities to brain tissue and maintains a constant micro-environment of the central nervous system (CNS). Disruption of the BBB by disease or drugs can compromise the CNS. Thus, agents that maintain BBB integrity may be powerful preventive and therapeutic approaches for neurological diseases including PD (Abbott et al., 2006; Sandoval and Witt, 2008; Zlokovic, 2008). BBB disruption is associated with astroglial activation, and the alteration of endothelial adhesive and tight junctional molecules (Abbott et al., 2006; Sandoval and Witt, 2008; Zlokovic, 2008). Many synthetic or natural agents, such as resveratrol and shikonin, block BBB disruption by reducing astroglial activation and the alteration of expression of junctional molecules (Lee et al., 2016; Zhao et al., 2017). In the present study, gintonin inhibited astroglial activation and blocked increase in the mRNA expression of endothelial adherens junctional molecules (ICAM-1 and VCAM-1) and in the SNpc and striatum following MPTP injection, while it prevented the decrease in that of tight junctional molecules (ZO-1 and claudin-3) (**Figure 5**). Taken together, that data indicate that gintonin could attenuate the degeneration of dopaminergic neurons caused by MPTP neurotoxicity, directly or indirectly mitigating BBB disruption. The cellular mechanism remains unclear.

Whether activation of LPA pathway protects dopaminergic neurons that are normally damaged in PD and whether gintonin could be critical role in the LPA pathway in PD are unclear. Here, although expression of LPARs was not investigated in all neural cell types in the SNpc and striatum following MPTP injection, the level of mRNA and protein expression of LPAR 1 and 3 was not significantly changed in the both tissues following MPTP injection, but their expressions were significantly increased by co-administration of gintonin with MPTP (**Figure 6**). Other types of LPAR did not show significant alteration. Currently, we failed to elucidate why gintonin administration enhanced the expression of only LPAR1 and 3 subtypes. It is possible that the gintonin-mediated anti-PD effects were mediated via LPAR1 and 3 in the brain rather than other subtypes in SNpc and striatum, based on the observation that gintonin-mediated anti-PD activity was blocked by Ki16425, an LPAR1/3 antagonist (**Figure 7**). Thus, the enhanced expression of LPAR 1 and 3 by gintonin in the presence of MPTP contributed to the protective effect against MPTP-induced neurotoxicity. However, further studies are needed to elucidate the precise molecular mechanisms underlying differential increases in brain LPAR1 and 3 levels following gintonin treatment in MPTP-induced PD animal model. Taken together, the present study showed that gintonin-mediated regulation of LPAR1 and 3 plays a key role in the amelioration of MPTP-induced SNpc and striatal toxicity.

Interestingly, multi-target effects of gintonin on MPTP neurotoxicity were neutralized by pre-treatment with Ki16425 (**Figure 7**). The results suggest that increased LPAR1 and 3 by gintonin directly or indirectly contributed to the protective effect against MPTP neurotoxicity via multiple targets, which was supported biologically. LPARs are widely expressed in neurons, microglia, astrocytes, and endothelial cells at a higher level in pathological conditions such as traumatic brain injury, neuropsychiatric disorders, and neuropathic pain (Crack et al., 2014; Yung et al., 2015; Velasco et al., 2017). LPA signaling stabilizes Nrf2 and increases the expression of genes (NQO1 and HMOX1) involved in oxidative stress response through LPAR1 (Venkatraman et al., 2015). Nrf2 modulation in response to NF-κB activation acts as a protective mechanism against inflammation (Wardyn et al., 2015). Taken together, the data support the suggestion that gintonin might contribute to anti-dopaminergic neuronal death via multi-target effects including anti-inflammation, anti-oxidant, and maintenance of BBB integrity through direct or indirect regulation of LPAR signaling pathway. Therefore gintonin may be exploited natural product-derived medication to prevent or treat PD via multi-target effects. The precise roles of gintonin on LPA pathway in the intact and diseased nervous system remain to be determined in the future.

## Conclusion

Most medications are available to control symptoms, because no innovative neuroprotective agents are yet available to treat multifactoral PD. Discovery of a multifunctional therapy targeting both symptomatic treatment and neuroprotection is a very attractive challenge to treat PD. Here, gintonin significantly inhibited the degeneration of dopaminergic neurons from MPTP-mediated neurotoxicity, possibly by multi-functional mechanisms including anti-dopaminergic cell death activity, anti-oxidative activity by stimulation of the Nrf2 pathway, anti-inflammatory activity by inhibition of the MAPKs and NF-κB pathways, and maintenance of BBB integrity, through the regulation of the LPA-LPARs signal pathway. Therefore, gintonin may be applied as natural product-derived multi-target drug to prevent and treat multifactoral PD.

## Author Contributions

JC performed the behavioral experiments, immunohistochemistry and Western blots, and prepared all figures. MJ carried out real-time PCR analysis and contributed to data interpretation. SO and S-YN contributed to draft of article and critical revision for important intellectual content. I-HC conceived all experiments, analyzed the results, and wrote the manuscript. All authors have read and approved the final manuscript.

## Conflict of Interest Statement

The authors declare that the research was conducted in the absence of any commercial or financial relationships that could be construed as a potential conflict of interest.

## Supplementary Material

FIGURE S1 Gintonin do not significantly affect LPAR2 and 4–6 signaling pathways in the SNpc and striatum after MPTP injection. **(A–H)** SNpc and striatum sample (*n* = 3 per group) from 7 days after MPTP-injection were quantified by real-time PCR to measure the alteration in expression of LPARs. mRNA expression of LPAR2 **(A,E)**, 4 **(B,F)**, 5 **(C,G)**, and 6 **(D,H)**. SNpc **(A–D)** and striatum **(E–H)**.

FIGURE S2 Original images of Western blots: Western blot analysis was performed using membrane strips containing specific proteins.
