# Supplementary figures and images for: Multi-Target Protective Effects of Gintonin in 1-Methyl-4-phenyl-1,2,3,6-tetrahydropyridine-Mediated Model of Parkinson’s Disease via Lysophosphatidic Acid Receptors
Source: Front Pharmacol. 2018 May 23;9:515. doi: 10.3389/fphar.2018.00515 (PMC5974039; doi:10.3389/fphar.2018.00515)

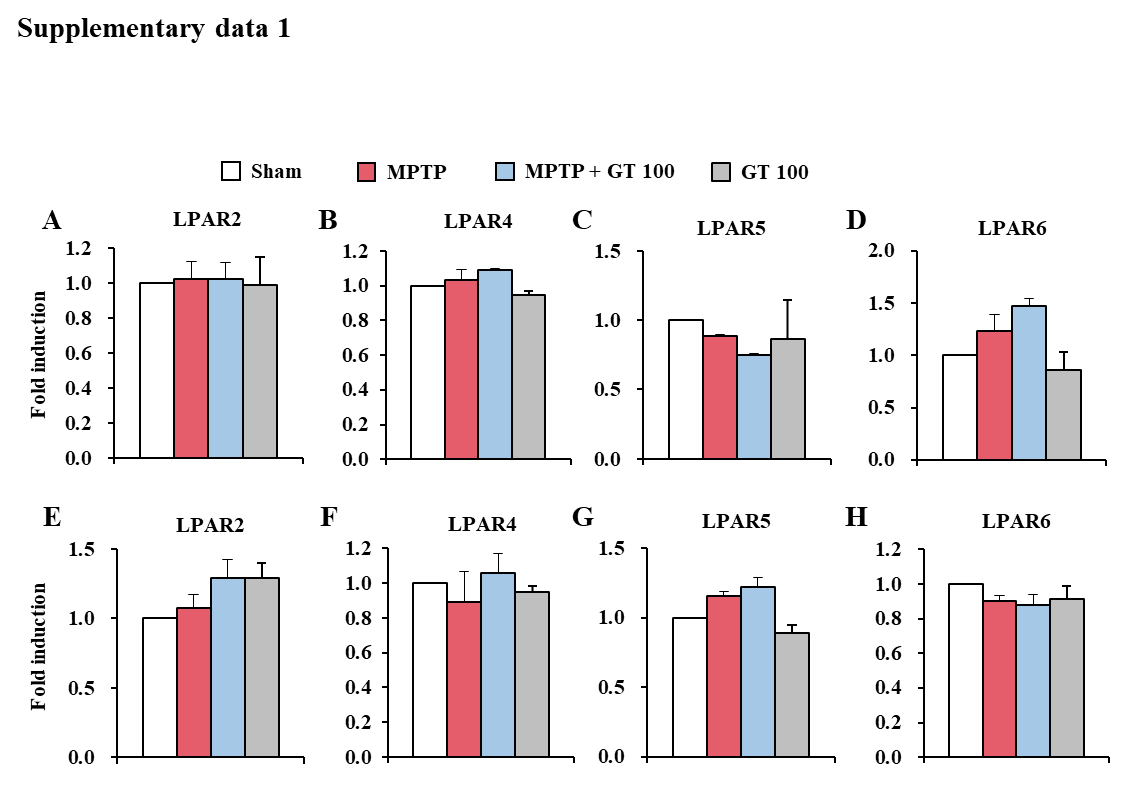

Supplement: FIGURE S1 — Gintonin do not significantly affect LPAR2 and 4–6 signaling pathways in the SNpc and striatum after MPTP injection. (A–H) SNpc and striatum sample (n = 3 per group) from 7 days after MPTP-injection were quantified by real-time PCR to measure the alteration in expression of LPARs. mRNA expression of LPAR2 (A,E), 4 (B,F), 5 (C,G), and 6 (D,H). SNpc (A–D) and striatum (E–H). [file Image_1.tif]

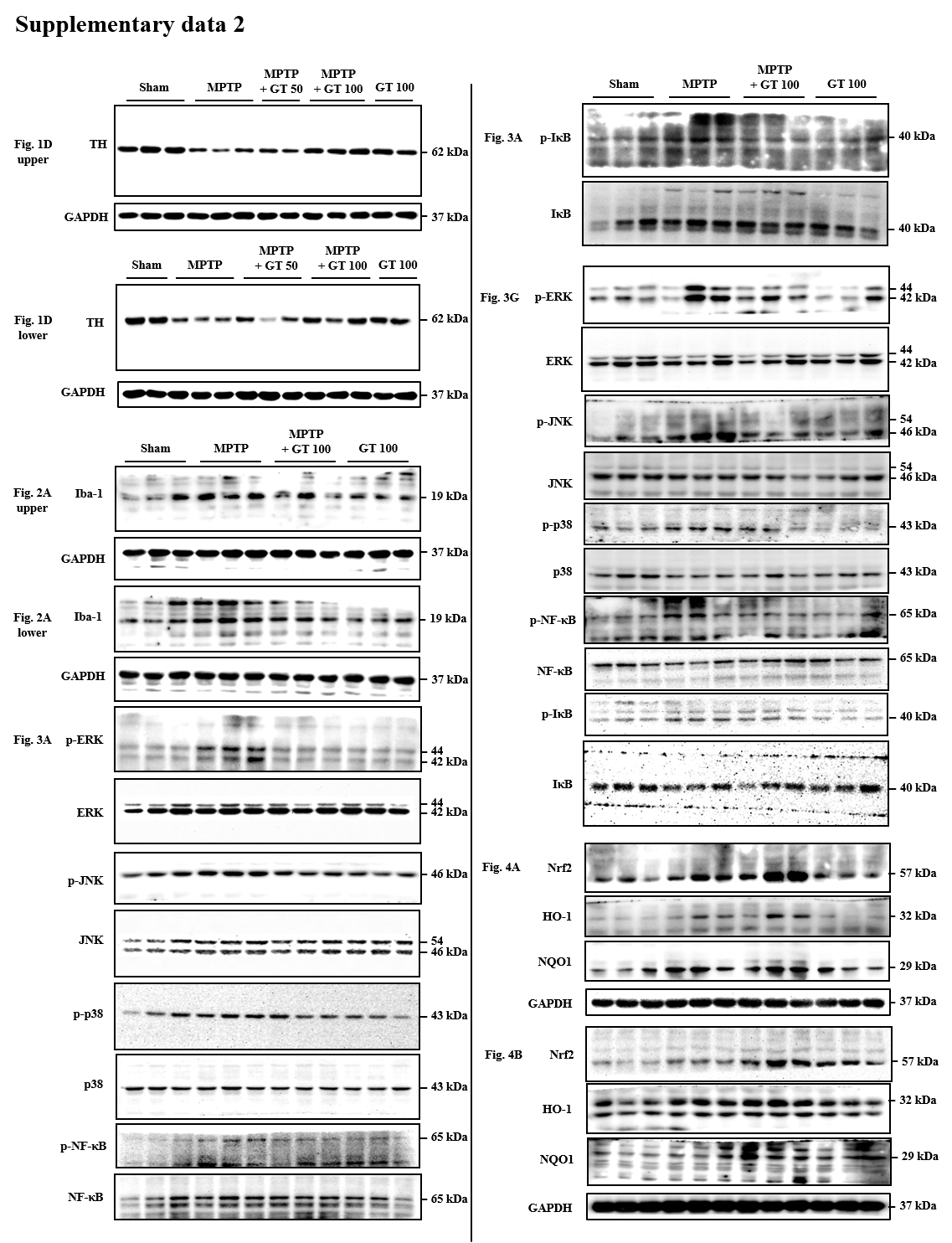

Supplement: FIGURE S2 — Original images of Western blots: Western blot analysis was performed using membrane strips containing specific proteins. [file Image_2.tif]

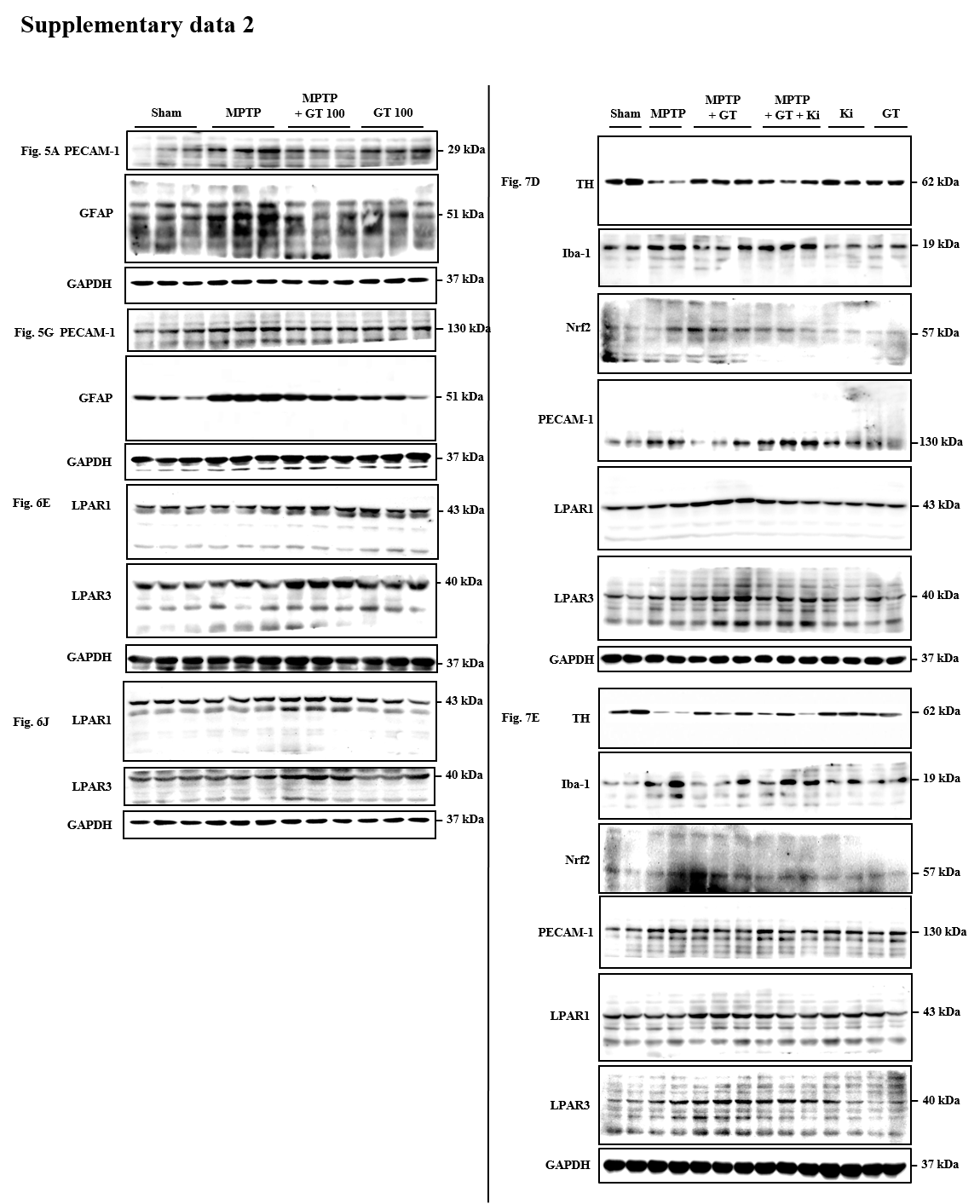

Supplement: Supplementary file 3 [file Image_3.tif]
